# Acknowledgement of manuscript reviewers 2014

**DOI:** 10.1186/s13006-015-0031-z

**Published:** 2015-02-07

**Authors:** Lisa H Amir

**Affiliations:** International Breastfeeding Journal, Melbourne, Australia

## Abstract

The editor of *International Breastfeeding Journal* would like to thank all our reviewers who have contributed to the journal in Volume 9 (2014).

**Clara Aarts**

Sweden

**Ojo Agunbiade**

South Africa

**James Akre**

Switzerland

**Alex Anderson**

United States of America

**Philip Anderson**

United States of America

**Annette Noble Beasley**

New Zealand

**Genevieve Becker**

Ireland

**Fetene Berhanu Belihu**

Australia

**Pamela Berens**

United States of America

**Colin Binns**

Australia

**Nancy Brent**

United States of America

**Wendy Brodribb**

Australia

**Amy Brown**

United Kingdom

**Miranda Buck**

Australia

**Susan Burger**

United States of America

**Marsha Campbell-Yeo**

Canada

**Ma del Carmen Casanovas**

Switzerland

**Caroline Chantry**

United States of America

**Elise Chapin**

Italy

**Donna Chapman**

United States of America

**Ellen Chetwynd**

United States of America

**Lumbwe Chola**

South Africa

**Anna Coutsoudis**

South Africa

**Meabh Cullinane**

Australia

**Marina Cuttini**

Italy

**Mary-Ann Davey**

Australia

**Emily de Jager**

Australia

**Tanya Doherty**

South Africa

**Susan Donath**

Australia

**Therese Dowswell**

United Kingdom

**Gudina Egata**

Ethiopia

**Megan Elliott-Rudder**

Australia

**Margaret Flood**

Australia

**Della Forster**

Australia

**Kevin Frick**

United States of America

**Judith Galtry**

New Zealand

**Ada Garcia**

United Kingdom

**Michel Garenne**

France

**Camila Giugliani**

Brazil

**Karleen Gribble**

Australia

**Luke Grzeskowiak**

Australia

**Rukhsana Haider**

Bangladesh

**Karin Hammarberg**

Australia

**Debra Hector**

Australia

**Caroline Homer**

Australia

**Bernardo Horta**

Brazil

**Kathryn Houk**

United States of America

**Russ Hovey**

United States of America

**Jenny Ingram**

United Kingdom

**Briana Jegier**

United States of America

**Kate Jolly**

United Kingdom

**Vishnu Khanal**

Nepal

**Hanne Kronborg**

Denmark

**Linda Kvist**

Sweden

**Miriam Labbok**

United States of America

**Laura Lamberti**

United States of America

**Rebecca Lander**

United States of America

**Ha Le**

Australia

**Cheryl Lovelady**

United States of America

**Nyovani Madise**

United Kingdom

**Cynthia Mannion**

Canada

**Susana Matias**

United States of America

**Ellen McIntyre**

Australia

**Helen McLachlan**

Australia

**Maureen Minchin**

Australia

**Elizabeth Moore**

United States of America

**Anita Moorhead**

Australia

**Pamela Morrison**

United Kingdom

**Melinda Munos**

United States of America

**Judith Myers**

Australia

**Nathan Nickel**

Canada

**Tinuade Ogunlesi**

Nigeria

**Funmilola OlaOlorun**

Nigeria

**Gillian Opie**

Australia

**Carmen Pallás**

Spain

**Tarané Probst**

Germany

**Phyllis Rippeyoung**

Canada

**Nikki Rogers**

United States of America

**Nigel Rollins**

South Africa

**Heather Rowe**

Australia

**Kath Ryan**

Australia

**Sina Safayi**

United States of America

**Jane Scott**

Australia

**Sonia Semenic**

Canada

**Touran Shafiei**

Australia

**James Shelton**

United States of America

**Ling Shi**

United States of America

**Rhonda Small**

Australia

**Zaharah Sulaiman**

Malaysia

**Kristin Svensson**

Sweden

**Kenzo Takahashi**

Japan

**Keiko Tanaka**

Japan

**David Tappin**

United Kingdom

**Gill Thomson**

United Kingdom

**Barbara Wilson-Clay**

United States of America

**Jane Yelland**

Australia

**Sera Young**

United States of America

**Irena Zakarija-Grkovic**

Croatia

